# Retraction: Berberine maintains the neutrophil N1 phenotype to reverse cancer cell resistance to doxorubicin

**DOI:** 10.3389/fphar.2023.1349235

**Published:** 2023-12-07

**Authors:** Frontiers Editorial Office

The journal retracts the 29 January 2020 article cited above.

Following publication, concerns were raised regarding the integrity of the images in the published figures. The authors failed to provide a satisfactory explanation during the investigation, which was conducted in accordance with Frontiers’ policies. As a result, the data and conclusions of the article have been deemed unreliable and the article has been retracted.

This retraction was approved by the Chief Editors of Frontiers in Pharmacology and the Chief Executive Editor of Frontiers. The authors agree to this retraction.

